# A New Work

**Published:** 1859-10

**Authors:** 


### A NEW WORK.

We have just received from the establishment of Rickey, Mallory & Co., a copy of Tome's Dental Surgery. We have not had time to examine it carefully, but from the character of the author and the hasty glance we have been able to take of a few pages, we hesitate not to pronounce it a good work, and one much needed by the profession, and especially by the students of the profession. As a text-book to the dental student, it will certainly be very valuable, and no student should be without it. It seems well written, and will certainly find a place in the library of every dentist.

After a careful perusal, we shall have more to say about it.

